# Age independently affects myelin integrity as detected by magnetization transfer magnetic resonance imaging in multiple sclerosis

**DOI:** 10.1016/j.nicl.2014.02.004

**Published:** 2014-03-31

**Authors:** R.D. Newbould, R. Nicholas, C.L. Thomas, R. Quest, J.S.Z. Lee, L. Honeyfield, A. Colasanti, O. Malik, M. Mattoscio, P.M. Matthews, M.P. Sormani, A.D. Waldman, P.A. Muraro

**Affiliations:** a Imanova Centre for Imaging Sciences, London, UK; b Division of Experimental Medicine, Imperial College London, UK; c Division of Brain Sciences, Imperial College London, UK; d Department of Imaging, Imperial College Healthcare NHS Trust, UK; e Neurosciences, GlaxoSmithKline Research and Development, UK; f Department of Health Sciences (DISSAL), University of Genoa, Italy; g Department of Clinical Neurosciences, Imperial College Healthcare NHS Trust, UK

**Keywords:** MTR, magnetization transfer ratio, NAWM, normal appearing white matter, NAGM, normal appearing gray matter, WM, white matter, GM, gray matter, WML, white matter lesion, GML, gray matter lesion, Magnetization transfer, MRI, Multiple sclerosis, Aging

## Abstract

**Background:**

Multiple sclerosis (MS) is a heterogeneous disorder with a progressive course that is difficult to predict on a case-by-case basis. Natural history studies of MS have demonstrated that age influences clinical progression independent of disease duration.

**Objective:**

To determine whether age would be associated with greater CNS injury as detected by magnetization transfer MRI.

**Materials and methods:**

Forty MS patients were recruited from out-patient clinics into two groups stratified by age but with similar clinical disease duration as well as thirteen controls age-matched to the older MS group. Images were segmented by automated programs and blinded readers into normal appearing white matter (NAWM), normal appearing gray matter (NAGM), and white matter lesions (WMLs) and gray matter lesions (GMLs) in the MS groups. WML and GML were delineated on T2-weighted 3D fluid-attenuated inversion recovery (FLAIR) and T1 weighted MRI volumes. Mean magnetization transfer ratio (MTR), region volume, as well as MTR histogram skew and kurtosis were calculated for each region.

**Results:**

All MTR measures in NAGM and MTR histogram metrics in NAWM differed between MS subjects and controls, as expected and previously reported by several studies, but not between MS groups. However, MTR measures in the WML did significantly differ between the MS groups, in spite of no significant differences in lesion counts and volumes.

**Conclusions:**

Despite matching for clinical disease duration and recording no significant WML volume difference, we demonstrated strong MTR differences in WMLs between younger and older MS patients. These data suggest that aging-related processes modify the tissue response to inflammatory injury and its clinical outcome correlates in MS.

## Highlights

• Magnetization transfer MRI was used in a cohort of 40 MS subjects differing by age.
• MTR metrics were different between MS groups and controls, as expected.
• MTR in normal appearing tissue did not differ between age-stratified MS groups.
• MTR in white matter lesions was strongly different between age-stratified MS groups.
• Results imply an age-related effect in tissue integrity in MR-visible lesions.

## 1 Introduction

Multiple sclerosis (MS) is a clinically heterogeneous disease most commonly presenting in young adults with a relapsing–remitting (RRMS) course. For the majority, the course of disease eventually becomes progressively disabling (secondary progressive, SPMS).

Natural history studies have demonstrated that age affects disease progression independent of disease duration (Koch et al., 2007; Scalfari et al., 2011). Latency to development of SPMS is reduced in older patients, with age of onset representing an independent predictor for time to progression (Koch et al., 2007). Indeed, the effects of age on progressive disability occur despite variation in the initial disease pattern preceding SPMS (Confavreux and Vukusic, 2006). Aging-associated delay in remyelination may underlie this phenomenon. Complete remyelination of gliotoxin-induced demyelination occurs faster in younger rats when compared with older rats (Shields et al., 1999) and by pairing their circulatory systems, it was demonstrated that exposure of older mice to the circulatory systems of younger mice led to a restoration of youthful remyelinatory potential (Ruckh Julia et al., 2012).

Although magnetic resonance imaging (MRI) has revolutionized clinical practice in MS (McDonald et al., 2001; Polman et al., 2011) parameters derived from conventional MRI have limited correlations with clinical measures of disability (Filippi et al., 1995a). While MRI is sensitive in identifying focal white matter MS lesions, conventional T1- and T2-weighted MR protocols cannot readily detect subtle changes in normal appearing white matter (NAWM) (Filippi and Agosta, 2007) nor cortical gray matter lesions, which have been demonstrated at post-mortem (Evangelou et al., 2000). Magnetization transfer (MT) parameters have been used to detect and quantify changes occurring outside lesions identified on conventional MRI. MT contrast represents proton interactions between free fluid and macromolecules, such as myelin (Filippi and Agosta, 2007) and provides a potential in vivo biomarker of ultrastructural integrity which is sensitive to pathology in vivo in a variety of neurological diseases, including MS. The most widely examined MT contrast parameter is the magnetization transfer ratio (MTR), representing the percentage reduction of MR signal when applying off-resonance radiofrequency irradiation. Reduced MTR suggests reduced exchange between macromolecular-associated and free water, most likely due to a reduction in the size of the macromolecular pool.

Mean MTR signals in NAWM are lower in MS patients, compared with controls (Filippi et al., 1995b), and MTR in the GM has been shown to predict disability progression (Agosta et al., 2006). The precise nature of tissue damage associated with MTR abnormalities is under contention; some studies have demonstrated strong correlations between myelin content and MTR (Schmierer et al., 2004), while others have demonstrated correlations between MTR and axonal density in both lesions and NAWM (van Waesberghe et al., 1999).

Here, in order to investigate the biological substrates underlying the clinical effects of age in MS, we examined to what extent CNS tissue injury differs amongst older and younger MS patients. We therefore selected two groups of MS patients differing by age but not by the duration of their disease and one healthy control cohort age-matched to the older MS patients to account for and interpret any age-related differences from normal aging. We examined the MTR distribution in visible white matter lesions and normal appearing gray and white matter.

## 2 Materials and methods

### 2.1 Patients

Forty patients with rapidly evolving MS enrolled in the Medical Research Council-funded Patient Research Cohort Rapidly Evolving Multiple Sclerosis study (PRC-REMS [http://clinicaltrials.gov/ct2/show/NCT01044576]) and thirteen healthy controls enrolled in GSK-sponsored EMI115241 were selected for inclusion. The studies had ethical approval from the London — Chelsea NRES Committee (NHS REC Ref. 09/H0708/61) and Essex 1 Research Ethics Committee (Ref: 11/EE/0026), respectively, and all subjects gave full informed consent in writing. All MS patients included had a diagnosis of MS according to the revised McDonald's criteria (Polman et al., 2011) with RRMS or SPMS, disease duration ≤ 5 years from clinical diagnosis, EDSS score 2.0–6.0 at screening and meeting criteria for highly active and/or treatment-refractory MS activity defined as: (a) Two or more clinical exacerbations in the previous 12 months, regardless of treatment; OR: (b) one clinical exacerbation and sustained increase in EDSS of at least 1 point in the previous 12 months after receiving, declining or not tolerating immune-modifying treatment, OR: (c) evidence of gadolinium (contrast)-enhancement or increase of T2 lesion load at MRI after receiving, declining or not tolerating immune-modifying treatment. Patients were assigned to one of two cohorts depending on their age: ‘young MS’ aged 25–35 years [n = 20] or ‘older MS’ 45–60 years [n = 20], both ranges inclusive. Healthy control subjects were included in the ‘older control’ cohort to age-match the ‘older MS’ group. Cohort demographics common for all three groups are reported in Table 1. MS-relevant characteristics for the two MS groups are reported in Table 2. At the blinded MRI data quality check, two patients from the ‘older MS’ group had excessive movement artifacts and two healthy control subjects had incidental findings. These four subjects were excluded from their respective groups prior to analysis.

### 2.2 MRI acquisition

**Fig. 1:**
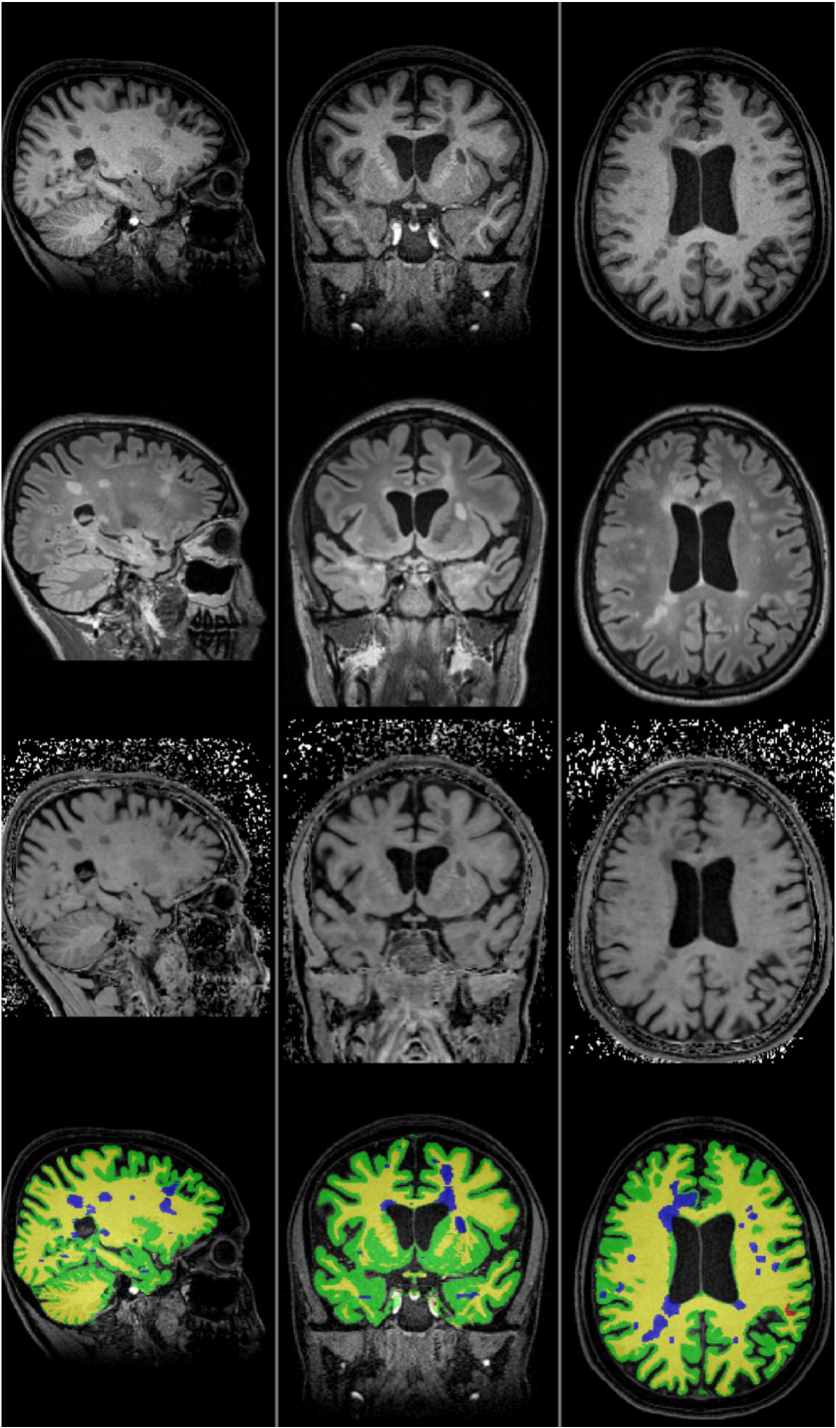
Representative images from a subject with a moderate lesion load. MPRAGE (top row), T2-w FLAIR (second row), resulting MTR map (third row), and ROI classifications overlaid on the MPRAGE (bottom row). Yellow = NAWM, green = NAGM, blue = WML, red = GML.

MRI images were acquired on a 3 Tesla Magnetom Verio scanner (Siemens Healthcare, Erlangen, Germany) at software version VB17 using a 12-channel phased array head coil with an 8-channel phased array neck coil. The following sequences were obtained in a single imaging session at a single site (Fig. 1).

Pre- and post-contrast T1-weighted 3D MPRAGE volumes were acquired based on the ADNI-GO recommended parameters (Jack et al., 2008): 256 × 192 mm field of view (FOV), 1 mm^3^ isotropic resolution, parallel imaging (PI) factor of 2, in 5 m: 21 s. Gadolinium injection (Gadoterate meglumine, Dotarem, Guerbet, 0.1 mmol/kg) was given < 5 min prior to the acquisition of the post-contrast volume.

A T2-weighted fluid-attenuated inversion recovery (FLAIR) 3D volume with 1 mm^3^ isotropic resolution for the delineation of white matter lesions was acquired, using a 3D T2w variable-refocusing angle TSE readout (Mugler and Brookeman, 2003). 160 sagittal sections in a single 3D slab were acquired with the following parameters: echo time (TE) 395 ms, repetition time (TR) 5 s, inversion time (TI) 1800 ms, 250 × 250 mm FOV, and a PI factor of 2 in 5 m: 52 s.

Magnetization transfer (MT) images were acquired using two pseudo proton density weighted (PDw) 3D spoiled gradient echo acquisitions (fast low angle shot (FLASH)). Common parameters include: 256 × 240 mm FOV, 192 sagittal sections per 3D slab, 1 mm^3^ isotropic resolution, parallel imaging factor of 2, TR of 27 ms with a flip angle of 5° in 7 m: 20 s, and 6 echoes acquired using 630 Hz/pixel bandwidth with TEs every 1.95 ms from 1.95 to 11.7 ms. Each high-bandwidth echo was summed to increase SNR without introducing off-resonance effects of low readout bandwidth (Helms and Dechent, 2009). One of these PDw volumes used an off-resonance MT pulse to add MT weighting (MTw), with a 12.24 ms duration Gaussian pulse at 2.2 kHz off resonance with a nominal flip angle of 540°.

All volumes were co-registered using the FSL Linear Image Registration Tool (FLIRT) (Jenkinson et al., 2002) to the MPRAGE volume to account for any movement between the acquisitions. MTR maps were calculated using the MTw and PDw acquisitions by the equation:

(1) $$\mathrm{MTR}=100\cdot \left(S_{\mathrm{PDw}}-S_{\mathrm{MTw}}\right)/S_{\mathrm{PDw}}.$$

### 2.3 MRI segmentation

Lesion segmentation in the MS cohorts was performed by a semi-automated thresholding technique with manual correction (Jim Version 6.0, http://www.xinapse.com/software.html) performed by a trained observer and corroborated by a second experienced observer, both blinded to age and clinical status. Areas were segmented from T1-weighted MPRAGE and T2-weighted FLAIR images to produce regions of interest (ROIs) representing white (WML) and gray matter lesions (GML). The FLAIR was used in conjunction with the MPRAGE due to advantages with respect to lesion conspicuity and detectability (Rydberg et al., 1994; Filippi et al., 1996).

Brain extraction and white/gray matter segmentation in all three groups was performed on T1-weighted images by an automated technique based on prior probabilities (Smith et al., 2002), subtracting the lesion masks from the tissue classifications in the MS groups to give normal appearing gray matter (NAGM) and white matter (NAWM). Segmentation masks were visually inspected by a trained observer, blinded to group, for assessment of the quality of segmentation.

### 2.4 Data analysis

Due to the small volume of the GML ROI, with a mean size of 96 mm^3^, and the low sensitivity of FLAIR to detect GMLs which may be a factor in the finding of GMLs in only 12 of 20 subjects in the younger MS group and 9 of 18 subjects in the older MS group, the GML ROI was excluded from further analysis. Each of the remaining three segmented tissue type masks were applied to the MTR maps to give both the ROI-based measure of mean MTR as well as to generate histograms of the MTR distribution in each ROI using 1 percent unit (p.u.) wide bins (van Buchem et al., 1996). Each histogram was normalized by dividing by the number of pixels in each histogram, to remove the influence of variation of brain and lesion volumes between subjects. For each distribution, the skew and kurtosis of the single modal distribution were calculated. Finally, the volume of each ROI was recorded.

Differences in MTR and ROI metrics and in demographic and clinical variables at the group level were assessed using the Wilcoxon rank sum test, except the gender, which used Fisher's exact test. Spearman's rank correlation coefficient was used to investigate associations between histogram metrics and functional status (EDSS scores) and the Pearson correlation coefficient for correlations between MTR metrics and brain volumes and log transformed lesion volumes. Multivariate logistic analysis was employed with MT variables input as individual predictors of age to distinguish which variables are independently correlated with the age group. The ability of the combination of the variables included in the multivariate model to distinguish younger and older patients was assessed by a ROC analysis and quantified by the area under the curve (AUC). A two-tailed *p*-value of < 0.05 was considered significant.

## 3 Results

### 3.1 Older patients have higher disability independent of other clinical variables

**Fig. 2:**
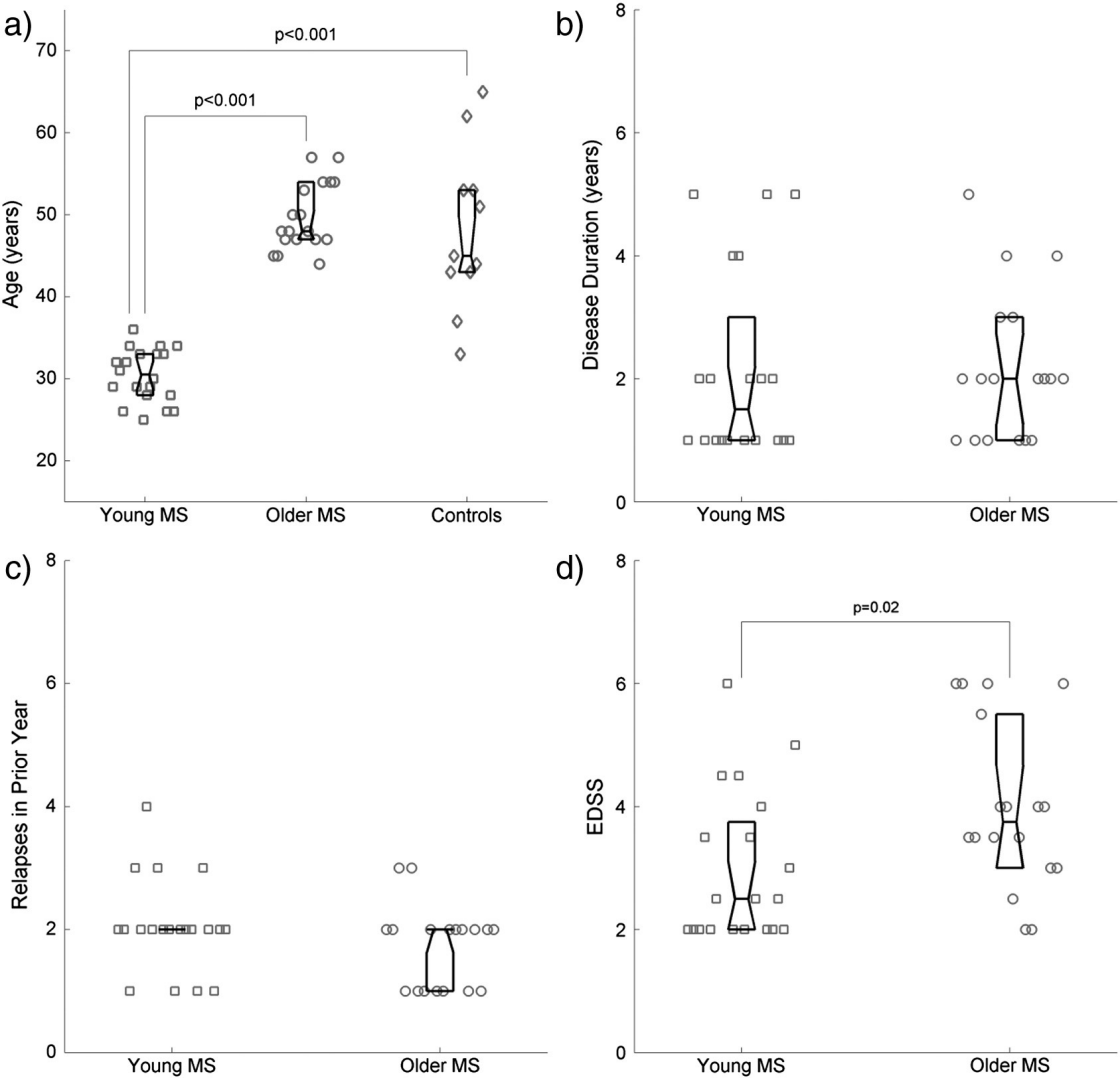
Age at MRI, disease duration, relapses, and EDSS across groups. Age at time of MRI (a) and clinical disease duration (b) were dictated by study criteria and are therefore strongly different (ages between younger and older groups) or well matched (all other comparisons). (c) No difference was found in number of relapses, but functional status as measured by EDSS (d) differs significantly between age groups.

The mean age at the time of MRI scan was 30.4 ± 3.3 years (mean ± SD) in the younger MS group, 49.7 ± 4.1 in the older MS group, and 48.0 ± 9.8 years in the older control group. The ages in the two older groups did not significantly differ, but were strongly different to the young group (Fig. 2a). Gender distribution was similar in all groups (Table 1). Though groups did not significantly differ for clinical disease duration (*p* = 0.59; Fig. 2b), recent relapses (*p* = 0.19; Fig. 2c) and previous or ongoing disease-modifying treatments, EDSS was significantly higher in the older group (younger group EDSS 2.5 (range 2–6), older group EDSS 3.8 (range 2–6); *p* = 0.019; Fig. 2d).

### 3.2 MTR measures

**Fig. 3:**
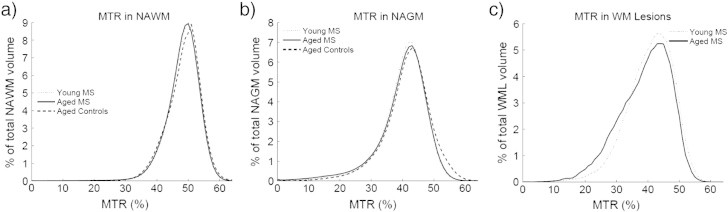
MTR histograms. NAWM shows a small leftward shift in both MS groups versus controls (a). NAGM has a more pronounced reduction in high MTR voxel counts (b). In WMLs, the older MS group shows much lower MTR values (c).

As seen in the rightmost column of Table 2, all the MTR measures were similar in the NAGM and NAWM in both MS groups. This stands in contrast to each MTR metric in the WML, which was significantly different between the young and older MS groups. The mean MTR was significantly lower in the older group (38.26 ± 2.16 p.u) versus the younger group (40.54 ± 1.94 p.u.) even though the number of white matter lesions and total WML volumes did not differ significantly. The median white matter lesion volume was greater in the younger group, but this did not reach significance as both groups had a large variability in the number and size of white matter lesions. The MTR distribution in WML can be seen in Fig. 3c to contain a higher proportion of low MTR voxels in the older MS group, thus altering the skew and kurtosis of these distributions.

Mean MTR was not different between controls and either MS group in NAWM, which excluded any MR-visible lesions. However, mean MTR was significantly different between controls and both MS groups in the NAGM. In both the NAWM and NAGM, the skew and kurtosis measures were significantly different from controls.

### 3.3 MTR parameters and disability can discriminate older and younger patient groups

**Fig. 4:**
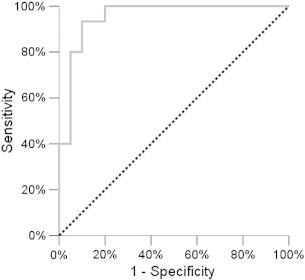
Multivariate analysis. ROC curve showing the ability of the combination of the 4 variables (EDSS, gray matter volume, T1 lesion volume, and mean MTR in WML) to distinguish young and old patients. AUC = 0.95, (95% CI = 0.88–1.00).

A multivariate logistic regression including the MTR parameters significantly different between age groups (mean MTR, skew, and kurtosis in WML) was run, adjusting for EDSS, brain volume, T2 and T1 lesion volumes. The final model (selected with a forward selection procedure) included EDSS (OR = 4.01, p = 0.011), gray matter volume (OR = 0.95, p = 0.001), T1 lesion volume (log scale) (OR = 0.10, p = 0.001) and mean lesion MTR (OR = 0.93, p = 0.03) as independent predictors of age group. This observation indicates that mean MTR in WML is significantly different between the two age groups also adjusting for other variables associated with age and disease (EDSS, brain and T1 lesion volumes). The area under the ROC curve (Fig. 4) derived from these 4 parameters' combination was 0.95 (95% CI = 0.88–1.00), indicating that this set of variables is effective at discriminating younger and older patients.

## 4 Discussion

In this study, brain MTR was compared between three groups: two MS groups differing only by age, and a control group that was age-matched to the older MS group. The MS cohort studied had a high disease activity. This cohort was chosen to allow a more robust investigation of lesion tissue as a larger number and/or volume of lesions was expected as compared to a less active or inactive group of patients. Further, any age-related differences in the degree of normal or lesional tissue injury would be more clinically relevant in such a population. After excluding abnormal tissue, the MTR distribution in NAWM and all MTR metrics in NAGM were found to be strongly different between controls and both MS groups, a similar result to many previous studies (Filippi and Agosta, 2007). Our study also showed similar MTR results in NAGM and NAWM in both the young and older MS groups, with no age-related effect. In strong contrast, MTR was significantly lower in WML tissue in the older MS group when compared with the young.

There have been several previous studies relating age to MTR in both MS and healthy populations. Rovaris et al. (2003) used whole-brain histograms to determine the average MTR, peak height, and peak position in a control population of 89 subjects with a mean age of 43.6 years (range 11–76). After excluding T2 hyperintensities, which correlated well with age, no age-related trends were found in any MTR measure. Mehta et al. (1995) used an ROI-based approach, and also saw no age effects in gray matter (GM) or white matter (WM) ROIs in 68 healthy subjects with an age range of 21 to 78. This contrasts with the healthy subject studies (51 subjects, mean age 55 years, range 21–77) of Hofman et al. (1999), who found an age-related decline in mean MTR in the WM, and Ge et al. (2002) (52 subjects, mean age 46 years, range 20–86), who found an age-related decline in several MTR histogram metrics in the WM and GM. However, neither study excluded abnormal areas defined on conventional MR images. Benedetti et al. (2006) reanalyzed the healthy subject data of Rovaris et al. (2003), splitting histograms into gray and white matter, and found significant correlations between age and MTR histogram parameters only for the GM, not for the NAWM. WM T2 hyperintensities were masked out before creating the MTR histograms for NAWM. After correcting for the number of T2 hyperintensities, gender, and tissue volume, no MTR measure remained significantly correlated with age. Silver et al. (1997) found small but statistically significant age-related declines in MTR in several WM ROIs in 41 healthy subjects (mean age 35.5 years, range 16–55) which excluded T2 hyperintensities.

In an MTR study of 30 primary progressive MS patients (mean age 40.7 years, range 25–51) and 30 healthy controls (mean age 39.4 years, range 27–53), Dehmeshki et al. (2003) found no correlation between age and MTR in any of the metrics in NAWM nor NAGM in MS patients, though the mean NAGM MTR was negatively correlated to age in controls. Finally, in an MTR study comparing 22 age-matched controls with 22 RRMS subjects with disease onset either before age 18 (mean age at scan 21.3, range 15–29) or after age 18 (mean age at scan 37.6 years, range 25–48), Oguz et al. (2010) found reduced MTR in the NAWM when compared to controls, but no age-related effect between the two MS groups.

Several conclusions can be drawn from this body of previous work. First, it is important to delineate visibly abnormal areas from normal appearing parenchyma when considering MTR metrics; the majority of studies that excluded visible lesions did not see age-related effects, while those that included them did, indicating that lesional MTR dominates these age-related effects. The study of Fazekas et al. (2005) showed an average 10% drop in mean MTR in white matter hyperintensities (WMH). The MTR in these WMH was not related to age, but as WMH become more prevalent with aging, this may explain much of the age-related MTR decline seen when not specifically excluding them from analysis. Though large, this effect is smaller than the difference seen between NAWM and WMLs in our MS groups of about 20%. The age-related effects in normal controls demonstrated in the two earlier studies underscore the requirement for an age-matched group, which has allowed us to decouple age-related changes from MS-related changes.

When comparing the control group to either MS group, strong changes in mean MTRs and distributions in the NAGM and MTR distributions in the NAWM were apparent. However, no significant differences were detected between the older and younger MS groups in NAWM and NAGM, which accords with the only previous study of aging and MTR in the MS population (Dehmeshki et al., 2003). Therefore, while MS-related changes were noted, no age-related changes were noted in the NAWM and NAGM.

This is the first study which has attempted to examine age-related changes within WMLs in MS. In WML, very strong differences in all MTR metrics were seen between the young and older MS groups, when controlled for clinical disease duration and despite finding similar lesion loads in both groups. This implies that age in MS patients exerts a direct negative effect upon CNS myelin integrity in MS WMLs that is reflected in MTR.

MTR parameters have been widely quoted within MS literature. In our study, average MTR values were within expected limits from previous studies; with average MTR of NAWM between 40 and 50 p.u. in a normal brain (Horsfield et al., 2003). We used semi-automated segmentation techniques with manual editing to delineate hyperintense lesions on T2-weighted images and hypointense lesions on T1-weighted images. This approach introduces a degree of inter-observer variability, and we have attempted to reduce resultant error by using a second experienced observer to corroborate lesion delineation. Automated methods of segmenting gray and white matter have been previously used in MS cohorts (Rovaris et al., 2003; Miller et al., 2002) with good reproducibility.

As is common practice in histogram analysis, the MTR histograms were normalized by each subject's tissue volume. However, the ROI volumes did differ in some comparisons. Even when WMLs and NAWM volumes were summed to compare total WM, MS subjects had less total WM than controls, indicating an expected pathological volume loss. Both MS groups had similar WM volumes. Interestingly, the young MS subjects had similar GM volumes as the older controls; the older MS subjects had less GM than either other group. Progressive GM loss is well described in MS (Valsasina et al., 2005); our data raise the possibility that this effect is more pronounced in older patients for a given clinical disease duration, but this would require a formal comparison including a young control group. As previously discussed, WML volumes were similar in both MS groups.

Our study has a number of limitations. Due to the lack of an available double inversion recovery (DIR) sequence (Wattjes et al., 2007) at the beginning of this study, GMLs were quantified on a 3D T2w-FLAIR volume, which has increased sensitivity to GMLs over a standard 2D spin-echo based approach, but less sensitivity than the 3D DIR sequence (Geurts et al., 2005). The delineation of GMLs was performed to ensure that NAGM was normal appearing on both T1w and T2w volumes. GMLs were found in 12 of the 20 and 9 of the 18 subjects in the young and older MS groups, respectively. If an additional 150% of the GMLs detected by 3D FLAIR would be classified as GML on DIR (Geurts et al., 2005), these additional lesions would be mostly included in the NAGM. This would represent 0.03% of the NAGM volume that was actually GML, and unlikely to affect the interpretation of NAGM.

Our cohort study included 40 MS subjects, similar to the cohort sizes in the previously discussed studies, but only 13 control subjects. Both our cohort sizes are smaller than other MTR studies in healthy subjects. As noted above, the chosen cohort of rapidly-evolving MS subject was expected to give a large effect size in MTR and lesion metrics between healthy controls and subjects with MS, mitigating the limitations of a small sample size. Indeed, the presence of benign or inactive forms of MS in an unselected cohort would have decreased the sensitivity of the experiment and hence the power to detect differences between younger and older patients. This small sample size did produce meaningful comparisons, though it was not large enough to interpret other variables, such as the underlying age-related decline in ROI volumes. Instead, these data must be interpreted using the results of other, larger studies.

One possible confounder when examining GM with MTR is partial volume effects between the GM and either or both cerebrospinal fluid (CSF) and WM. CSF has a low MTR, and therefore MTR would decrease in nominally GM voxels which contain CSF, while the higher MTR in WM would increase the effect seen in nominally GM voxels which contain WM. This study used high resolution (1 mm isotropic) 3D datasets, which will reduce the partial volume effect, and a high threshold (0.75) for confidence in the automated tissue classification. GM atrophy would increase the degree of partial volume effects, and therefore might give spurious MTR in atrophied GM. However, MTR metrics were strongly different between controls and young MS groups and NAGM volume was similar, obviating these concerns.

We have attempted to match disease duration between groups by defining it as clinical disease duration based on the time elapsed since clinical diagnosis of MS. This may not reflect the biological duration of the disease, and there is a theoretical risk that older individuals have had a longer subclinical duration before diagnosis. If the onset of symptoms could be reliably determined, it may be more closely linked to biological onset, but to our knowledge no evidence exists that latency from biological to clinical onset is longer in patients diagnosed with MS at an older age.

This histogram-based study removed any anatomical location information of the lesions, and treated the entire identified lesion as a uniform area in the analysis. A more refined approach, stratifying by brain region or subdividing lesions into perilesional and deep lesional areas may show further effects; however, this was not done in this study.

To our knowledge, no previous study has compared MTR amongst MS groups with age as the primary variable. While many studies have examined the relationship of MTR values with clinical variables, we have focused on the effects of age on the brain in MS. We suggest that the decreased efficiency of myelination in aging, well described in nonhuman primates (Bowley et al., 2010), and demyelinating injury in multiple sclerosis both contribute to the additional loss of myelin integrity in WMLs revealed by MTR in the older patients in our study. This pathology may be underpinned by a relative failure of remyelination in the older patient; by analogy with animal studies where younger rats remyelinate more quickly than in older rats (Shields et al., 1999), and defective remyelination is abrogated by exposing older mice to the circulation of younger animals (Ruckh Julia et al., 2012).

In support of our interpretation of the MTR results and of data from experimental models, we find that current age predicts functional disability, as measured by EDSS, when patients are matched for clinical disease duration and do not differ in other disease-relevant variables such as number of preceding relapses and disease-modifying treatments. This finding is in line with large natural history studies demonstrating that older patients reach the progressive stage of the disease more quickly than younger patients (Koch et al., 2007; Scalfari et al., 2011). Therefore the reduction in MTR seen in the older age group might be better explained as a modifying effect of aging on the outcome of focal WM inflammatory insult in MS (which may include decreasing efficiency of repair with aging) rather than by the severity of the primary inflammatory insult, as exemplified in animal studies (Shields et al., 1999; Ruckh Julia et al., 2012).

In conclusion, the results of our MTR analysis imply that aging in the context of MS plays an important role in brain white matter integrity and this is mirrored by differences in functional status between age groups despite matching of the salient clinical variables. This work corroborates earlier findings that indicate the crucial role that aging plays in the clinical trajectory of MS, which is of relevance to the MS community for the understanding and development of treatments for progressive forms of the disease.

## Disclosure statements

Dr Newbould was an employee of GlaxoSmithKline and held stock options in GlaxoSmithKline at the time of the study.

Dr Thomas, R Quest, Dr Lee, and L Honeyfield report no disclosures.

Dr Malik has received honoraria and travel bursaries from Biogen Idec and Novartis.

Dr Mattoscio has received travel funding from Biogen Idec.

Prof. Matthews is a part-time employee of GlaxoSmithKline Research and Development and receives funding from the UK Medical Research Council.

Dr MP Sormani has received consulting fee or honoraria from Biogen IDEC, Merck Serono, Actelion, Synthon, and Allozyne.

Dr Waldman has received educational grants and speaker honoraria from Bayer HealthCare.

Dr Muraro has received travel support and speaker honoraria from Bayer HealthCare, Bayer Pharma, Biogen Idec, Merck-Serono and Sanofi Aventis.

## Figures and Tables

**Table 1:** Demographics of study population.

	‘Older control’	‘Young MS’	‘Older MS’	P (oC/yMS)	P (oC/oMS)	P (yMS/oMS)
Age (years)	48.5 (9.0)	30.4 (3.3)	49.7 (4.1)	**< 0.001**	0.30	**< 0.001**
Age range (years)	37–65	25–36	44–57			
Sex (f/m)	8/3	17/3	13/5	0.63	1.0	0.44
MS subtype		20 RRMS	16 RRMS; 2 SPMS			0.42
Disease duration (mean yrs ± SD)		2.2 ± 1.5	2.2 ± 1.2			0.59
EDSS (median, range)		2.5 (2–6)	3.75 (2–6)			**0.019**
Prior year clinical relapses (mean ± SD)		2.0 ± 0.7	1.7 ± 0.7			0.19
Disease modifying treatment (n treated patients) At any time		9 (3 Avonex, 2 Betaferon, 3 Copaxone, 1 Rebif)	12 (3 Avonex, 2 Betaferon, 3 Copaxone, 3 Rebif, 1 azathioprine)			
On-going at time of scan		8 (2 Avonex, 2 Betaferon, 3 Copaxone, 1 Tysabri)	7 (1 Avonex, 2 Betaferon, 2 Copaxone, 2 Tysabri)			

All values are reported with the standard deviation in parentheses. Legend: Older controls vs. young MS (oC/yMS), older controls vs. older MS (oC/aMS), young MS vs. older MS (yMS/oMS). Bold typeface indicates a P value less than 0.05.

**Table 2:** MTR measures between groups.

	ROI	Older control	‘Young MS’	‘Older MS’	P (oC/yMS)	P (oC/oMS)	P (yMS/oMS)
Mean (p.u.)	NAWM	48.30 (0.87)	48.32 (1.26)	48.01 (1.26)	0.964	0.505	0.452
	NAGM	41.27 (0.74)	40.16 (1.19)	39.84 (1.25)	**0.009**	**0.002**	0.424
	WML	–	40.54 (1.94)	38.26 (2.16)	–	–	**0.0025**

Skew	NAWM	− 0.873 (0.132)	− 1.213 (0.338)	− 1.277 (0.344)	**0.0035**	**0.00095**	0.563
	NAGM	− 0.820 (0.124)	− 1.122 (0.187)	− 1.216 (0.139)	**< 0.00001**	**< 0.00001**	0.089
	WML	–	− 0.839 (0.361)	− 0.570 (0.306)	–	–	**0.026**

Kurtosis	NAWM	5.421 (0.534)	8.689 (2.757)	8.826 (2.362)	**0.00058**	**< 0.00001**	0.87
	NAGM	4.747 (0.394)	5.577 (0.775)	5.524 (0.819)	**0.0025**	**0.0068**	0.83
	WML	–	4.392 (1.396)	3.241 (0.797)	–	–	**0.0074**

Volume (cc.)	NAWM	604.16 (72.71)	525.87 (52.23)	524.70 (62.28)	**0.00097**	**0.0042**	0.947
	NAGM	572.5 (52.23)	559.10 (46.28)	522.63 (62.28)	0.55	**0.037**	0.082
	WML	–	10.57 (11.34)	6.54 (6.65)	–	–	0.196

All values are reported with the standard deviation in parentheses. Legend: Older controls vs. young MS (oC/yMS), older controls vs. older MS (oC/aMS), young MS vs. older MS (yMS/oMS). Bold typeface indicates a P value less than 0.05.
